# Modifications to the Aesop's Fable Paradigm Change New Caledonian Crow Performances

**DOI:** 10.1371/journal.pone.0103049

**Published:** 2014-07-23

**Authors:** Corina J. Logan, Sarah A. Jelbert, Alexis J. Breen, Russell D. Gray, Alex H. Taylor

**Affiliations:** 1 SAGE Center for the Study of the Mind, Department of Psychological and Brain Sciences, University of California Santa Barbara, Santa Barbara, California, United States of America; 2 School of Psychology, University of Auckland, Auckland, New Zealand; 3 School of Psychology and Neuroscience, University of St Andrews, St Andrews, United Kingdom; Centre national de la recherche scientifique, France

## Abstract

While humans are able to understand much about causality, it is unclear to what extent non-human animals can do the same. The Aesop's Fable paradigm requires an animal to drop stones into a water-filled tube to bring a floating food reward within reach. Rook, Eurasian jay, and New Caledonian crow performances are similar to those of children under seven years of age when solving this task. However, we know very little about the cognition underpinning these birds' performances. Here, we address several limitations of previous Aesop's Fable studies to gain insight into the causal cognition of New Caledonian crows. Our results provide the first evidence that any non-human animal can solve the U-tube task and can discriminate between water-filled tubes of different volumes. However, our results do not provide support for the hypothesis that these crows can infer the presence of a hidden causal mechanism. They also call into question previous object-discrimination performances. The methodologies outlined here should allow for more powerful comparisons between humans and other animal species and thus help us to determine which aspects of causal cognition are distinct to humans.

## Introduction

Humans have an excellent understanding of the relationships between cause and effect [1]-[3]. Currently, little is known about which aspects of this understanding are unique to our species or how such understanding evolves, despite a number of recent claims for human uniqueness [2], [4]. One reason for the slow progress in this area is that, until recently, the predominant test of causal cognition was the trap-tube task, where food has to be moved out of a horizontal tube while avoiding a hole [5]–[11]. There are a number of issues with this paradigm [12]–[13], including the failure of critical controls by adult humans [14], the large effect that small modifications to the paradigm have on problem-solving capabilities [7], and the inability of large numbers of individuals from each species tested to learn any strategy to solve the task [5]–[6], [11].

The Aesop's Fable paradigm has recently emerged as an alternative test of causal cognition [15]. This paradigm is useful because it requires a novel form of tool use (stone dropping into water-filled tubes), which is not seen in the wild and thus allows cross-species comparisons of causal cognition [16]. In this paradigm, subjects are presented with a water-filled tube that contains an out-of-reach floating food reward. To solve this problem, subjects must drop objects into the tube to raise the water level, thus bringing the food within reach. Rooks (*Corvus frugilegus*), Eurasian jays (*Garrulus glandarius*), New Caledonian crows (*Corvus moneduloides*) and children (*Homo sapiens*) can discriminate between functional and non-functional substrates (water vs. sand/sawdust/air) and objects (large vs. small, sinking vs. floating, hollow vs. solid) when presented with choices [13], [15]–[18]. However, these species differ in their reactions to the U-tube task, a variant of the Aesop's Fable paradigm. This task examines how subjects respond to an unexpected effect after dropping a stone into a tube, namely that the stone causes water to rise in a seemingly unconnected adjacent tube.

Eurasian jays were the first species presented with the U-tube task, in an attempt to determine whether a confusing cause and effect relationship would inhibit their learning of a simple rule [18]. In the task, two large tubes were positioned adjacent to a small middle tube containing food. Since the small tube was too small to drop stones into, subjects had to drop stones into one of the large tubes. Only one of the large tubes was connected to the small tube under the table, thus making this tube the functional option, though because the base was covered, the causal mechanism was hidden. Each large tube was marked with a distinct color cue. To solve the task, subjects had to notice that dropping a stone into a tube marked with one color resulted in the rise of the floating food in the middle tube. None of the jays solved this task, indicating that a confusing causal relationship appears to inhibit the learning of a simple associative rule: ‘drop the stone in the red tube’. All New Caledonian crows [13] and all 5-year-old children also fail this task, though all children 7 years and older solve it [16]. One explanation for the bird's failure is that the distance between the two large tubes was smaller than in the other tasks, which might have interfered with successful discrimination [19]. In other water tube discrimination experiments that involve two tubes, such as the sand vs. water experiment, the tubes are positioned 30 cm apart [13], [15]–[18]. In the U-tube experiment, the two large tubes are only 15 cm apart [13], [16], [18]. It could be that this reduction in distance made it more difficult for the birds to inhibit switching between tubes and so prevented discrimination. A further issue with the current U-tube methodology is that, even if subjects succeed, such as children 7 to 10 years of age, there are two competing hypotheses for their performance. One possibility is that they have inferred the presence of a hidden causal mechanism, namely the connection between the two tubes. Another possibility is that the subjects are highly sensitive to perceptual feedback [16], [18], [20]–[21], and thus notice the effect stone dropping has on the adjacent small tube.

An additional limitation of previous studies concerns the ability of crows to make functional object discriminations. While current results suggest the crows make discriminations based on the causal properties of the objects involved, an alternate possibility is that they discriminate simply because they prefer to handle one object type over another. In particular, preferences could be driven by familiarity. For example, crows could prefer to handle solid rather than hollow objects simply because solid objects look more similar to the stones they have previously dropped in the tube and encountered in the wild. While simple preferences for approaching previously rewarded objects were controlled for in one study [17], no study has so far examined if subjects have a preference for handling certain objects.

Given the history of the trap-tube task, where small modifications to the procedure such as letting subjects pull rather than push the food leads to large behavioral differences [7], it is critical that the effect of modifications to the Aesop's Fable task be explored. Here, we use the Aesop's Fable paradigm to test causal cognition in New Caledonian crows. This species may have sophisticated causal cognition in the wild since it makes and uses tools to extract hidden food [22] and appears to make inferences about hidden causal agents [23]. We addressed the limitations of the U-tube and object preference tasks outlined above. First, we replicated the object and substrate experiments in Jelbert and colleagues [13] to ensure that our results were consistent with results from previous studies on New Caledonian crows. We also replicated Jelbert and colleagues' [13] volume discrimination experiment. However, instead of providing the crows with 12 objects, which allowed individuals to obtain food from either tube if they persisted long enough, we provided the crows with only enough objects (four) to correctly solve the task by choosing the more efficient tube. We then addressed the methodological limitation of the U-tube experiment by increasing the distance between large tubes, such that it was identical to the distance used in standard tube discrimination experiments. Additionally, we gave each wide tube its own narrow tube to make the distinction between the two sets of tubes clear. To investigate whether object preferences are actually related to the functionality of the task, we presented crows with hollow and solid objects of the same weight when the task required an object with a particular weight, rather than a particular displaceable volume. Finally, to test whether New Caledonian crows make inferences about a hidden causal mechanism, we designed a novel uncovered U-tube task to be given after subjects had attempted to solve the original U-tube task. We removed all arbitrary cues and exposed the hidden mechanism, the connecting pipe between two of the tubes. If subjects successfully solved the original covered U-tube task by inferring that the large and small tube were connected, we expected them to subsequently choose the visibly connected large tube rather than the unconnected one. However, if they had simply learned the association between the arbitrary color cue and the movement of the food, then their learning should be disrupted by the removal of this cue.

## Methods

### Ethics Statement

This research was carried out in accordance with the University of Auckland's Animal Ethics Committee (permit number R602). All aspects of the study protocols were approved as part of this permit.

### Subjects

Eight wild-caught New Caledonian crows (2 adults, 6 juveniles; 3 females, 5 males) were studied from May to July 2013 in an aviary on Grand Terre, New Caledonia. Crows were housed in 2×3×3 m aviaries, had *ad libitum* access to water, and were fed dog food, papaya, eggs, and meat daily. Sex was estimated from body weight [24] and age was determined from mouth coloring. Birds were tested individually and in visual isolation of others. If a bird was not participating in a trial, bait was placed on the table to encourage participation. In experiments where two tubes were involved, the bait was located halfway between the tubes and birds were given up to 30 seconds to inspect the tubes before the objects were placed on the table. Birds had access to four stones, weighing 14–17 g and displacing 4–5 mm of water, in each experiment except sinking vs. floating, solid vs. hollow, and wide vs. narrow. The object discrimination and wide vs. narrow experiments followed the methodology of Jelbert and colleagues [13]. Twenty trials were conducted for each experiment. Experiments 1 through 7 were carried out in sequential order on all of the birds, with some of the birds experiencing experiment 8 before experiment 1 as noted in the section for experiment 8.

### Stone Dropping

**Figure 1:**
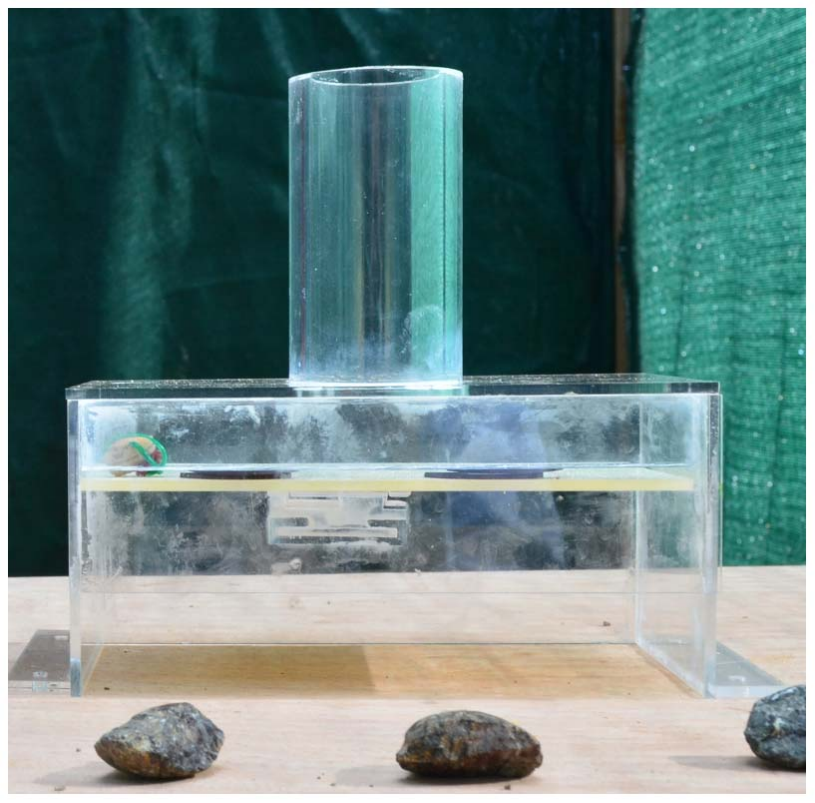
Single stone dropping apparatus.

Before participating in this experiment, birds were trained to drop stones onto a platform, which collapsed to release a food reward. The apparatus was a clear cast acrylic box (180×110×85 mm) with a 90 mm tube (outer diameter = 51 mm, inner diameter = 40 mm) on top and a platform inside held up by a magnet. When a stone was dropped through the tube and onto the platform, the platform collapsed and the meat sitting on top of the platform fell out (Figure 1). Four stones were provided for this task. Once birds began stone dropping, they were given a further 24 trials on this task before proceeding to multi-stone training.

### Multi-stone Training

**Figure 2:**
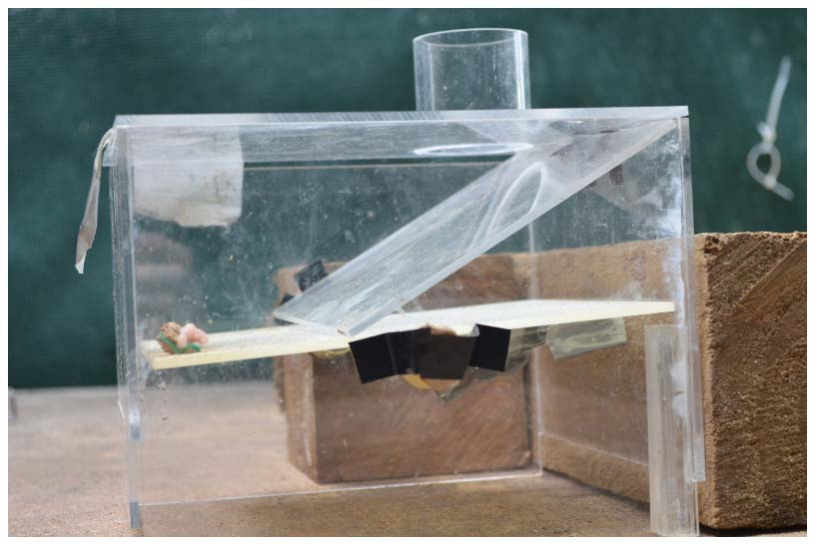
Multi-stone dropping apparatus.

After having successfully conducted the single stone dropping trials, each bird carried out 10 multi-stone dropping trials where they had to drop several stones in each trial onto a platform to receive the food. The apparatus consisted of a platform balanced on a stick inside a clear cast acrylic box (200×180×150 mm) with a 50 mm tube (outer diameter = 50 mm, inner diameter = 44 mm) on top of the box (Figure 2). The platform tipped and the food fell out when 2–4 stones were dropped down the tube because there were counterweights on the opposite side of the platform. This apparatus trained the crows to drop multiple stones to gain meat. Seven birds passed the multi-stone training and one (Kitty) skipped this training due to experimenter error and went straight to water vs. sand. Six birds completed 10 trials proficiently and one bird (Buster) required 32 trials to become proficient at this task.

### Reachable Distance

The reachable distance is defined as the water level at which the bird can reach the floating food (meat cube attached to cork) with its bill and was measured from the base of the cast acrylic tube to the top of the floating food inside the tube. We determined the reachable distance by presenting the bird with a tube filled with usually 120 mm of water. If the bird could reach the food, the water level was lowered until their reachable distance was known. During the experiment, 12 mm was subtracted from their reachable distance such that the food would be out of reach enough to require dropping 2–4 stones to bring the food within reach. Reachable distances were calculated for each bird for the standard tube and small tube (in the U-tube experiments), and the average reachable distance (128 mm) as found by Jelbert and colleagues [13] was used for the wide and narrow tubes.

### Experiment 1: Water vs. Sand

**Figure 3:**
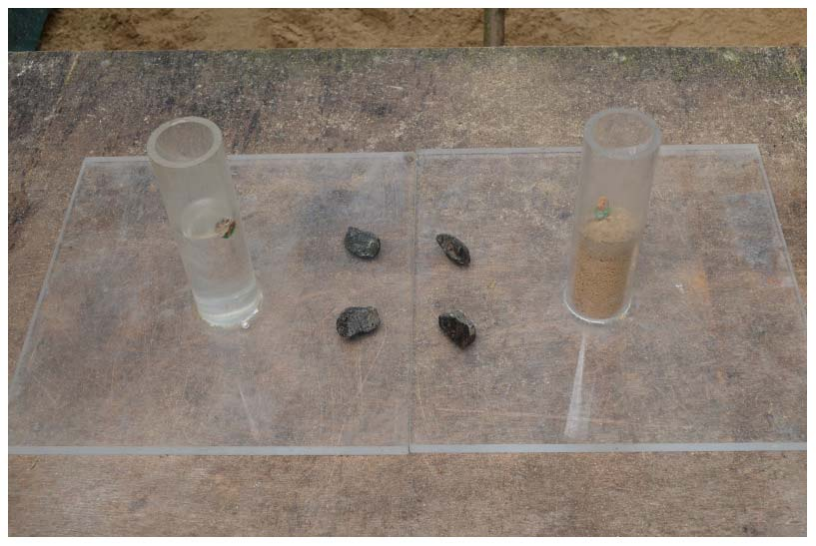
Water vs. sand experimental set up.

**Figure 4:**
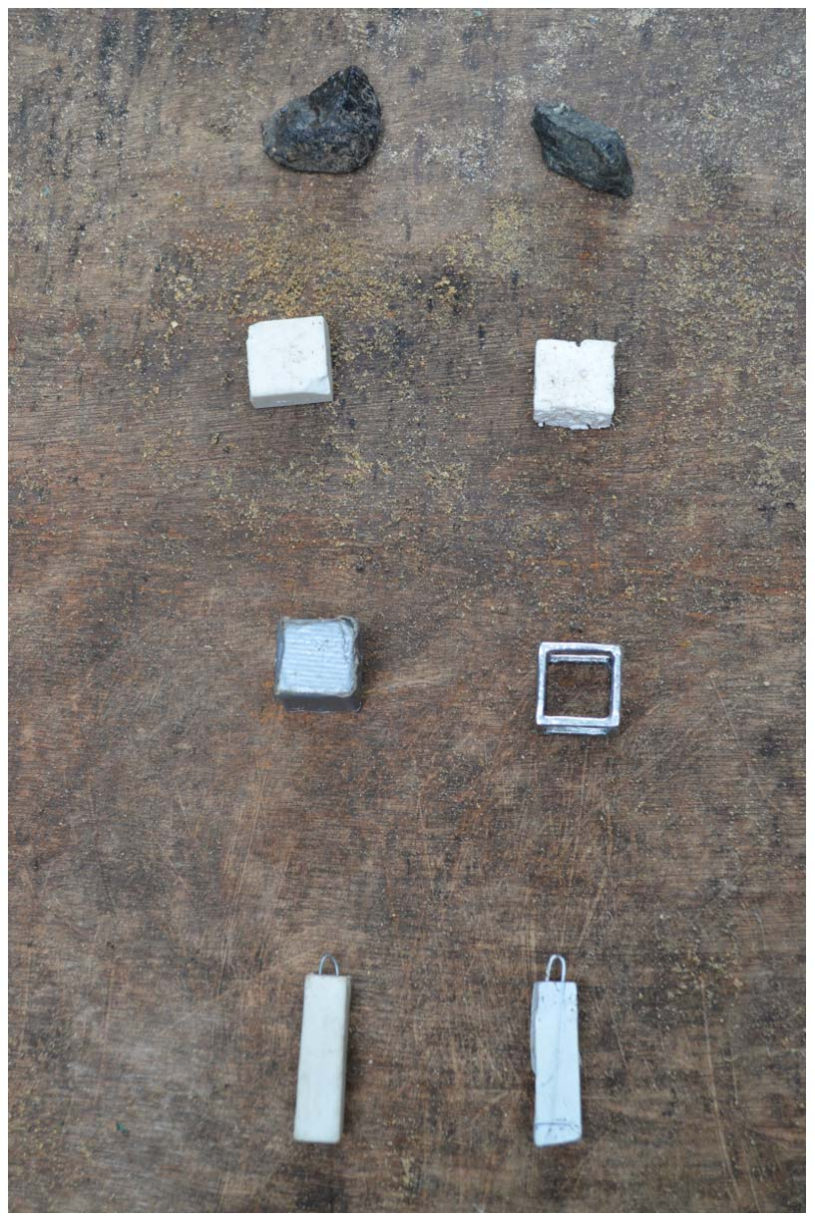
Objects used: top pair = stones, second pair = eraser/polystyrene for sinking vs. floating, third pair = solid/hollow, bottom pair = eraser/fimo clay for wide vs. narrow.

Each tube was clear cast acrylic with an outer diameter of 51 mm, inner diameter of 40 mm, and height of 170 mm, and was glued to a 300×300×12 mm clear cast acrylic base. Birds were given 10 habituation trials for the sand (non-functional) and water (functional) tubes by presenting the tubes in a pseudorandomized order (alternating sides for the first two trials, presenting the same tube on the same side up to two times consecutively). The tube openings were taped shut and meat cubes were placed on top and at the base of each tube. The first tube from which a bird ate food was noted to evaluate whether the bird showed a preference. If an obvious preference developed as habituation trials progressed, more meat was placed on the least preferred tube to reduce the preference, to ensure the bird attended to the functional properties of the task when the experiment began. If a bird did not confidently approach the tubes within 10 trials, the taped tubes were placed in their home aviary, meat was periodically placed on the tubes until the bird habituated, and then another 10 habituation trials ensued. After habituation, the experiment began and the sand and water tubes were pseudorandomized for side. Four stones were placed between the tubes with two sitting on the left tube's base and two sitting on the right tube's base (Figures 3 and 4).

### Experiment 2: Sinking vs. Floating

**Figure 5:**
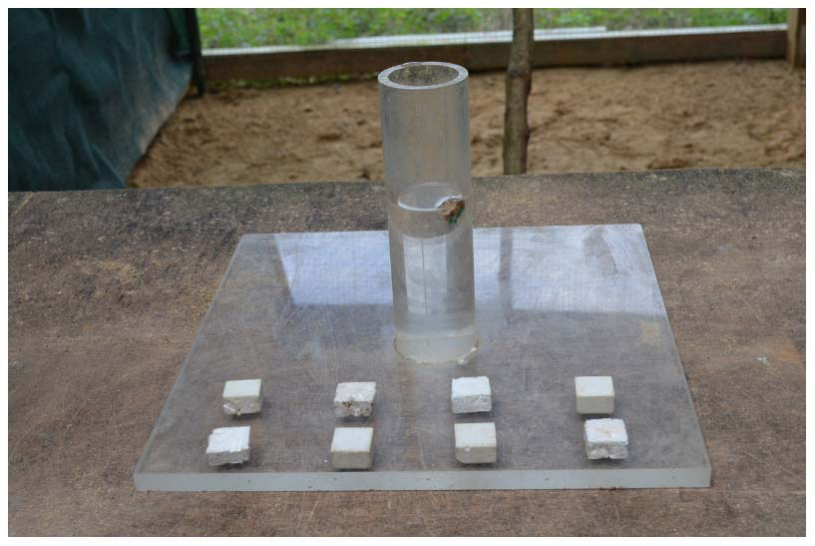
Sinking vs. floating experimental set up.

The functional sinking objects were erasers (9 g, 20×20×10 mm) that displaced 3.5 mm of water in the standard tube and the non-functional floating objects were polystyrene blocks (0 g, 20×20×10 mm) that displaced 0.3 mm of water. Birds were habituated to the objects by giving them three trials where one sinking and one floating object were placed on the table and meat was put on top and below the objects so the bird had to interact with them to get the food. The first object touched during a habituation trial was noted to document preferences. In the experiment, we gave birds four erasers and four polystyrene objects placed in pseudorandomized pairs in front of the tube (Figure 5).

### Experiment 3: Solid vs. Hollow

The more functional solid objects were metal cubes that were empty inside (9 g, 20×20×20 mm) and displaced 6 mm of water and the less functional hollow objects were metal cube frames that lacked sides (9 g, 20×20×20 mm) and displaced 1 mm of water. Birds were habituated to the objects using the same method as in the sinking vs. floating experiment. We gave birds four solid and four hollow objects arranged as in the sinking vs. floating experiment.

### Experiment 4: Wide vs. Narrow Equal Water Levels

**Figure 6:**
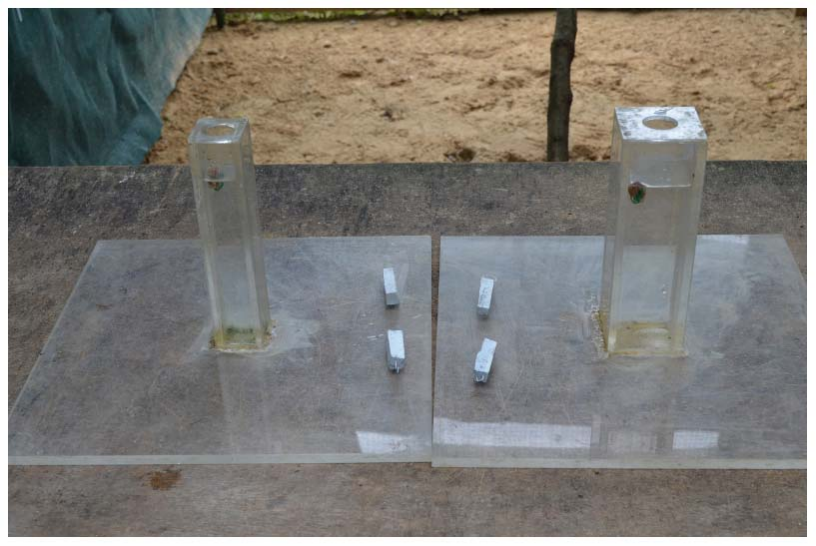
Narrow and wide tubes with equal water levels.

In this experiment, the narrow tube was the functional tube because the water levels in both tubes were set to the narrow tube's reachable distance. Since the wide tube required more objects to make the water rise the same distance, the food would not come within reaching distance even if all four objects were dropped in. The narrow (24×24×174 mm, volume = 100,224 mm^3^) and wide (44×44×174 mm, volume = 336,864 mm^3^) tubes were made out of clear cast acrylic sheets and glued to 300×300 mm bases (Figure 6). Both tubes had clear cast acrylic lids with holes of the same diameter (25 mm) to equalize how far the bill could reach through the hole into the tube. The relative differences in volume between the standard cylindrical water tube (213,628 mm^3^) and the wide and narrow tubes were roughly equivalent (wide tube = 123,236 mm^3^ larger than standard tube and narrow tube = 113,404 mm^3^ smaller than standard tube). Birds had access to four objects of the same type (8–10 g erasers that displaced 9 mm in narrow or 1 mm in wide tube, or 5–7 g fimo clay cuboids that displaced 9 mm in narrow or 3 mm in wide tube; both objects were 40×10×10 mm) with metal handles on one end. We switched from erasers to clay objects because Damien began eating the erasers. Therefore, Q, 007, Kitty, and Lady had erasers for objects, while Damien and Buster had clay objects. Objects were placed between the tubes as in the sand vs. water experiment.

### Experiment 5: Wide vs. Narrow Unequal Water Levels

**Figure 7:**
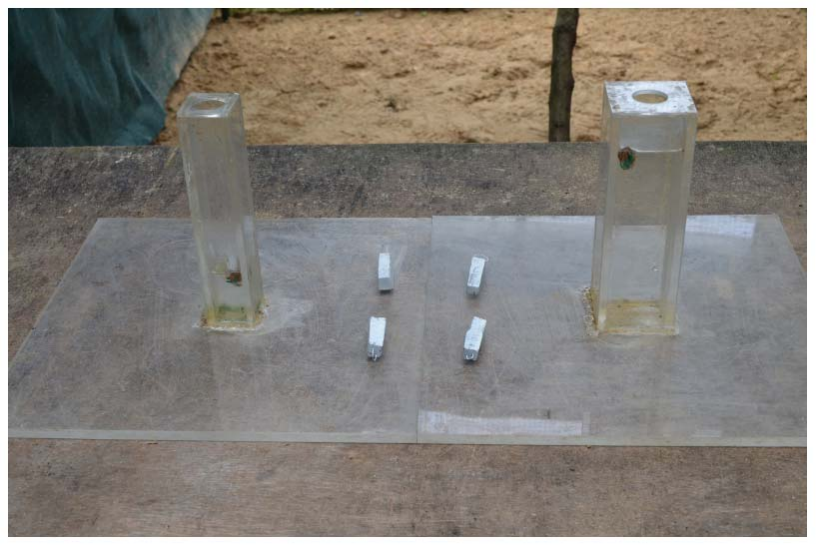
Narrow and wide tubes with unequal water levels.

The water level in the narrow tube was completely unreachable at 50 mm, regardless of how many objects were dropped in, while the water level in the wide tube was set to a distance such that dropping the objects in would raise the food within reach (Figure 7). Birds that passed the wide vs. narrow equal water level experiment were given this experiment to determine whether they were able to switch their preference when the opposite tube becomes more functional.

### Experiment 6: Colored U-tube

**Figure 8:**
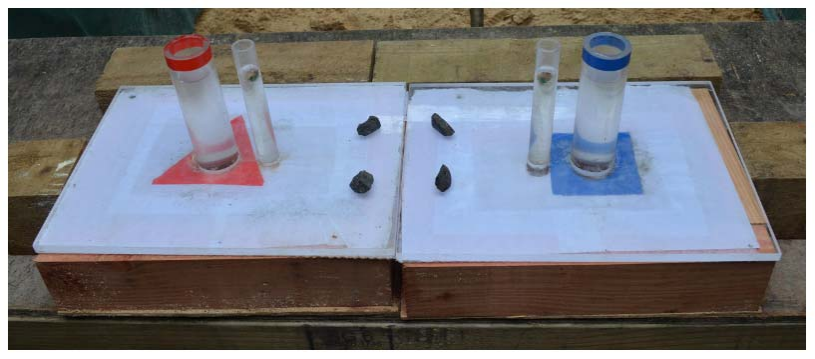
Colored U-tube experimental set up.

This experiment consisted of two apparatuses, each containing a standard tube and a small tube (small tube: outer diameter = 25.4 mm, inner diameter = 19 mm) 25 mm apart, with 170 mm sticking out and 80 mm below a clear cast acrylic lid (300×400×12 mm) on a wooden box (Figure 8). The lid was covered with paper to prevent subjects from observing the tubes underneath. The small tubes contained food. Stones were too large to drop into the small tubes; they could only be dropped into the standard tubes. Under the lid, one apparatus had a tube (outer diameter = 25.4 mm, inner diameter = 19 mm) that connected the standard and small tubes such that when stones were dropped into the standard tube, the water in the small tube would also be displaced, raising the food reward. The other apparatus did not have a connector tube, thus it was non-functional. Each apparatus, the connected or non-connected, was marked with red or blue tape at the top and base (red triangle or blue square on the paper cover over the lid) of the standard tube to indicate which apparatus was connected. The apparatuses were oriented with the small tubes nearest each other, making the distance between them 300 mm, and the connected and non-connected apparatuses were pseudorandomized for which side they were placed on. Birds were habituated to the two apparatuses in the same way as in the sand vs. water experiment (all tubes were taped over the top and had equal water levels), and their reachable distances for the small tube were carried out with the standard tube water level matching that of the small tube. We gave birds access to four stones lined up between the apparatuses as in the sand vs. water experiment.

### Experiment 7: Uncovered U-tube

**Figure 9:**
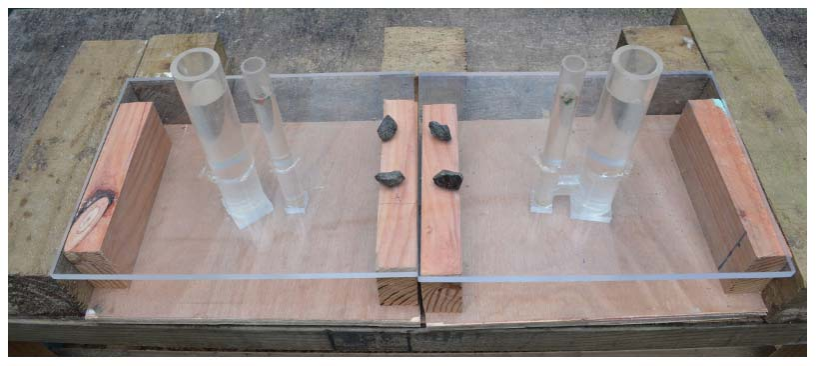
Uncovered U-tube experimental set up.

This experiment was the same as the colored U-tube experiment, except that we removed the colored tape, the paper cover over the lid, and the front and back of the wooden box so the bird could see which apparatus was connected (Figure 9).

### Experiment 8: Solid vs. Hollow on the Multi-stone Platform

This was the same as the solid vs. hollow experiment with the water tube, except that instead of a water tube, the multi-stone platform was used. Since both object types weighed the same, they were equally functional on the platform, whereas the solid was the functional object in the water tube since it displaces more water. Half of the birds were given this experiment before having experience with water tubes and half after the solid vs. hollow experiment with the water tube to determine whether they attended to the functional properties of the objects.

### Side Bias

If, during the experiments involving two tubes, a bird showed a side bias, the trials were paused and 5–20 trials of color choice were given, after which the experiment was restarted. The color choice test draws their attention to color rather than space and can break their pattern of always choosing one side without attending to the details of the task. In the color choice test, birds were presented with two PVC tubes (diameter = 32.5 mm, length = 45 mm glued to two 45×45 mm pieces of plywood): one gold tube that always contained meat and one silver tube that never contained meat. The tubes were placed, left side first, in a pseudorandomized order on the table and oriented such that the bird could not see into the tube without approaching it. The first tube approached and looked into was considered the chosen tube and once the bird left that tube, the trial was stopped. All birds were trained on the color choice test to an accuracy of at least 17 successful trials out of 20 before beginning the water tube experiment.

### Analysis

The exact binomial test was applied to the first choice in first trials for all birds in experiments 1–3, 4–5, and 6–7, and to all correct choices for each bird in each experiment at the 20-trial level to determine whether there were significantly more successes than predicted by chance (probability of 0.5) using the statistical software R [25]. P-values within each hypothesis were adjusted to correct for type 1 errors from conducting multiple tests using the Holm-Bonferroni method (R package: stats, function: p.adjust, method: Holm). A Generalized Linear Mixed Model (GLMM) was used to determine whether performance, the percentage of correct choices per bird per trial (response variable), changed across the 20 trials (trials grouped to determine where learning occurred: 1–5, 6–10, 11–15, and 16–20) in any given experiment (explanatory variables; see Table S1.1 in File S1). The GLMM had a Poisson distribution and log link (R package: lme4 [26]), and bird was included as a random factor to account for repeated tests on each subject. The response variable was categorical, thus did not need to adhere to a normal distribution. The top model was selected by comparing all possible model combinations according to their Akaike weight (scale: 0–1, all models sum to 1) using the dredge function in the R package MuMIN [27]. The top model was only considered reliable if its Akaike weight was 0.90 or higher [28].

## Results

See (Tables S2.1–2.8 in File S2) to see the order in which each choice was made for all trials in all experiments.

### Experiment 1: Water vs. Sand

Three birds passed this experiment by dropping stones into the water-filled tube significantly more than the sand-filled tube (see Holm-Bonferroni corrected binomial test p-values per bird in Table 1). Q made only three errors, which occurred in his first six trials. Two birds failed and three did not complete it. Of those that did not complete it, two were removed from the experiment because they would not drop stones into water. Of all of the stones that were dropped by the five birds that participated in this experiment, 74% were dropped into the water-filled tube. No birds showed a preference for either tube during the habituation trials (Holm-Bonferroni corrected binomial test: p = 1.00 for every bird, n = 8 birds).

**Table 1:** Summary of which birds passed which 20-trial experiments according to the Holm-Bonferroni corrected binomial test (p values listed in each cell).

Bird (color bands)	Age	Water vs. Sand	Sink vs. Float	Solid vs. Hollow	Wide vs. Narrow Equal	Wide vs. Narrow Unequal	Colored U-tube	Uncovered U-tube
Q (YG)	Juvenile	<0.001	0.004	<0.001	0.02	0.02	0.74	0.2
007 (Y)	Juvenile	<0.001	0.004	<0.001	1.00	–	0.08	1.00
Trooper (BO)	Juvenile	X	–	–	–	–	–	–
Kitty (WR)	Juvenile	0.08	<0.001	<0.001	0.03	0.3	0.04	1.00
Candy (WO)	Juvenile	X	–	–	–	–	–	–
Lady (YR)	Adult	<0.001	<0.001	<0.001	0.02	<0.001	0.74	1.00
Buster (OO)	Adult	X	<0.001	<0.001	<0.001	<0.001	X	–
Damien (WLB)	Juvenile	1.00	<0.001	<0.001	1.00	–	0.15	1.00
		n = 5	n = 6	n = 6	n = 6	n = 4	n = 5	n = 5

X  =  did not complete this experiment, –  =  did not participate in this experiment.

### Experiment 2: Sinking vs. Floating

Six out of six birds passed this test by choosing the sinking objects significantly more (85% of the time across all birds) than the floating objects (Table 1). Damien and Buster made no mistakes and Lady made only four errors, which occurred in her first three trials. Across all birds, there were no preferences for either object during habituation trials (uncorrected binomial test: p = 0.81, n = 6 birds).

### Experiment 3: Solid vs. Hollow

Six out of six birds passed by choosing the solid objects significantly more (95% of the time across all birds) than the hollow objects (Table 1). 007, Kitty, Lady, and Buster made no mistakes and Damien made only two errors, however no bird made an error until trial eight. Across all birds, there were no preferences for either object during habituation trials (uncorrected binomial test: p = 0.39, n = 7 birds).

### Experiment 4: Wide vs. Narrow Equal Water Levels

Four birds passed this test by dropping objects into the narrow tube significantly more (64% of the time across all six birds) than in the wide tube, and two birds failed (Table 1, Video S1). There were no preferences for either tube during habituation trials (uncorrected binomial test: p = 1.00, n = 2 birds).

### Experiment 5: Wide vs. Narrow Unequal Water Levels

Of the four birds that passed experiment 4, three of them also passed the unequal water level experiment by dropping significantly more objects into the wide tube (79% of the time across all birds), and one bird failed (Table 1). Buster was error-free and Lady made only four mistakes.

### Experiment 6: Colored U-tube

One bird, Kitty, passed this test by dropping stones significantly more into the connected apparatus, while four birds failed and one stopped participating (the connected tube was chosen 47% of the time across all birds; Table 1, Video S2). No birds showed a preference for either the red or blue tube during the habituation trials (Holm-Bonferroni corrected binomial test: p = 1.00 for each bird, n = 6 birds).

### Experiment 7: Uncovered U-tube

Five out of five birds failed this test by dropping stones randomly into either the connected or the non-connected apparatus (the connected tube was chosen 44% of the time across all birds; Table 1). Birds rarely bent over to inspect the part of the apparatus that was newly exposed, which included the connector tube.

### Experiment 8: Solid vs. Hollow on the Multi-stone Platform

Three birds (Trooper, Lady, and Damien) experienced the platform experiment before the water tube experiment, two birds (007 and Kitty) experienced the platform experiment after the water tube experiment, and one (Buster) experienced only the platform experiment. All birds in both experiments dropped significantly more solid objects than hollow objects into water tubes and platforms regardless of their prior experience (20-trial Holm-Bonferroni corrected binomial test p-values for the platform experiment: Trooper<0.001, Lady<0.001, Damien<0.001, 007<0.001, Kitty<0.001; 95% of objects dropped across all birds were solid).

### First choices in first trials

Birds chose the correct substrate (water) and object (sinking and solid) as their first choice in their first trial significantly more than expected by chance when combining data from experiments 1–3 (uncorrected binomial test: p = 0.002, 15 correct choices out of 17 total choices, n = 5 birds in experiment 1, n = 6 birds in experiments 2 and 3). Experiments 1–3 tested similar types of preferences and were combined to increase the sample size for the binomial test. Regarding volume discriminations, the correct volume was not chosen significantly more than chance when combining results from experiments 4 and 5 (uncorrected binomial test: p = 1.00, 5 correct choices out of 10 total choices, n = 6 birds in experiment 4, n = 4 birds in experiment 5). The correct tube in the U-tube experiments was not chosen significantly above chance when combining data from experiments 6 and 7 (uncorrected binomial test: p = 1.00, 5 correct choices out of 10 total choices, n = 5 birds in experiments 6 and 7).

### Did performance increase with an increasing number of trials?

Performance was influenced by experiment and trial and by an interaction between experiment and trial as evidenced by the fact that the best model of all possible model combinations was the full model (performance ∼ experiment * trial + (1|bird), Akaike weight = 1). Performance increased with an increasing number of trials in all experiments, except in solid vs. hollow, where the best performances were in the first five trials, and the uncovered U-tube where performance decreased as trials increased, potentially due to their lack of motivation to complete this difficult and unrewarding experiment (Table 2). Performance increased for trials 11–20 in water vs. sand, sinking vs. floating, and both wide vs. narrow experiments, indicating that birds learned about the task primarily during the first 10 trials. While learning occurred during the first five trials and trials 11–15 of the U-tube experiment, it was not sufficient for most birds to pass this test.

**Table 2:** The best fitting generalized linear mixed model (GLMM) examining the effect of experiment and trial on performance (percent correct choices per bird per trial) with bird as a random factor (variance and standard deviation for the random factor are reported in the estimate and SE columns respectively).

Factor	Trials	Estimate	SE
Intercept (U-tube)	1–5	3.90	0.04
U-tube	6–10	0.27	0.04
	11–15	0.06	0.04
	16–20	0.18	0.04
Water vs Sand	1–5	0.46	0.04
	6–10	−0.26	0.05
	11–15	−0.08	0.05
	16–20	−0.07	0.05
Sinking vs Floating	1–5	0.53	0.04
	6–10	−0.26	0.05
	11–15	0.06	0.05
	16–20	−0.04	0.05
Solid vs Hollow	1–5	0.71	0.03
	6–10	−0.30	0.05
	11–15	−0.11	0.05
	16–20	−0.22	0.05
Wide vs Narrow Equal	1–5	0.25	0.04
	6–10	−0.27	0.05
	11–15	0.19	0.05
	16–20	0.02	0.05
Wide vs Narrow Unequal	1–5	0.36	0.04
	6–10	−0.26	0.05
	11–15	0.14	0.05
	16–20	0.01	0.05
Uncovered U-tube	1–5	0.19	0.04
	6–10	−0.27	0.05
	11–15	−0.23	0.06
	16–20	−0.58	0.06
Random effect: Bird		0.005	0.07

SE = Standard Error.

## Discussion

As in past studies, New Caledonian crows preferred to drop stones into water-filled, rather than sand-filled tubes, and they preferred to drop objects that sank or were solid, rather than floating or hollow objects [13], [17]. However, while past work suggested that New Caledonian crows cannot discriminate between water-filled tubes of different volumes [13], here three of the six birds tested not only preferred to drop stones into a narrow tube, but then switched their preference to a wide tube when the water level in this tube was significantly higher. This switch from the narrow to the wide tube shows that the success in the equal water level condition is not simply due to these three crows having a preference for narrow tubes. While previous work has suggested that New Caledonian crows [13], like Eurasian jays [18], and children under 7 years of age [16], cannot solve the U-tube task, here one crow, Kitty, was able to do so by learning the connection between the arbitrary color cue and the movement of the food. However, all crows, including Kitty, subsequently failed our novel U-tube task with the exposed connection, which suggests that Kitty's success on the colored U-tube was due to the color cue association and not to the inference of a hidden causal mechanism or attention to the movement of the food. Crows also showed a preference for solid, rather than hollow, objects of the same weight when presented with a task not involving water, which suggests that they may have a bias for solid objects independent of the task at hand.

In first trials, significantly more correct choices were made in the substrate and object discrimination experiments, but not in the narrow vs. wide equal water levels, colored U-tube, and uncovered U-tube experiments. The crows' lack of an initial preference for particular tubes or objects during habituation suggests that first trial performance was not based on a bias to approach specific objects. In the experiments where first choices on first trials were correct, aside from the solid vs hollow condition, crows showed a learning effect in the first 10 trials, with performance improving in trials 11-20. This suggests that, while they had high overall levels of success across 20 trials, the crows needed experience with the objects, substrates, and tubes to settle on the functional option. These results are consistent with previous water tube experiments where rooks, Eurasian jays, and other New Caledonian crows showed some learning effects across the first five trials [13], [15], [17]–[18].

While our results show that the crows did not have a bias to approach objects to gain food, they may have had a bias towards the type of objects they preferred to pick up, given that all three birds without water tube experience chose solid rather than hollow objects of the same weight when presented with a platform apparatus. This finding calls into question the previous object discrimination choices of children [16], jays [18], and New Caledonian crows [13], [21]. The possibility that, irrespective of the task at hand, subjects have preferences for particular objects (*i.e.*, solid and sinking objects), particularly those that look more like objects they have previous experience with (*i.e.*, stones), has not been sufficiently well controlled for in past studies. Interestingly, it was only in the solid vs. hollow condition that no learning effect was shown, which suggests that the crows may have had a specific bias for solid objects, rather than a more general bias for the functional objects in our experiments. However, further work is required to test this hypothesis. It is also important to note that a handling preference has not yet been controlled for in children [16], and it may explain children's performances to date.

Our results differ from previous findings where no crows passed the wide vs. narrow equal water level experiment [13]. It appears that providing enough objects (four rather than twelve) for the bird to succeed only in the narrow tube, but not in both of the tubes, provided the motivation to choose the more efficient tube. That three birds then preferred a wide tube when the water level was unequal shows that these crows made discriminations based on water volume because they switched their preference from the narrow to the wide tube, indicating that they did not simply prefer to drop objects into the narrow tube. This finding raises two possibilities. The first is that crows are able to imagine changes in the magnitude of a causal relation. The crows only had prior experience dropping stones into a tube of one volume to raise its water level. They could have used their knowledge of the relationship between this tube size, the object, and the water level to mentally model the effect on the water level of dropping the same object into a bigger or smaller tube. This would have led them to choose the narrow tube. In contrast to this high level explanation, an alternate possibility is that the crows attended to differences in the feedback generated by dropping objects into the narrow tube (the floating reward moved significantly closer to the top of the tube) and the wide tube (minimal movement occurred). Further testing is required, using a tube where visual feedback is not available, to distinguish between these causal relation and feedback explanations

The performance of Kitty in solving our modified U-tube apparatus raises the possibility that past failures by corvids [13], [18] may be due to problems with tube discrimination when the stone dropping tubes are close together. Kitty's successful solution of this problem suggests two hypotheses. Like 42% of children between ages 4 and 10 [16], Kitty may have inferred the presence of a hidden causal mechanism linking the two tubes of the U-tube task. Alternatively, consistent with the most likely explanation for children's success on this task, she may have paid more attention to feedback than the other crows: by carefully watching the effect of a stone drop into each tube she may have noticed which tube caused the water level in the small tube to rise. However, these hypotheses both predict that Kitty should have passed both U-tube experiments. Though Kitty chose the correct tube on the first trial of the uncovered U-tube experiment, she did not consistently choose the connected tube. Thus, rather than inferring the presence of a hidden causal mechanism or attending to feedback, Kitty may have instead associated the color cue with the eventual receipt of the food. Further testing is required, given that children have not yet been tested on the novel uncovered U-tube task used here. Past research has shown the pitfalls of assuming how humans will react when faced with causal problems [29].

Despite the last 20 years of work on what animals understand about their physical world, we still have a rudimentary understanding of the cognition behind how animals solve problems. One of the key issues in the past has been the lack of rigorous testing of the experimental methodologies themselves, particularly the relation between small changes in procedure and animals' performance, which has resulted in the formation of strong conclusions about issues, such as human uniqueness, before the hypotheses under examination have been thoroughly tested [2], [6]–[7], [10]. The results here highlight a number of methodological limitations in the Aesop's Fable paradigm. With appropriate modifications to this paradigm, it should be possible to overcome these issues in future studies and so produce useful results for comparative cognition research. Comparisons will be particularly interesting between corvids and apes as they will provide insights into how the very differently structured bird and mammalian brains understand the causality of the world.

## Supporting Information

File S1 **The percentage of correct choices per bird per trial per experiment.**
(PDF)

File S2 **The order in which each choice was made for all birds in all trials in all experiments.**
(PDF)

Video S1 **Wide vs. narrow equal water levels: Buster trial 2.**
(MP4)

Video S2 **Colored U-tube: Kitty trial 4.**
(MP4)
