# Supplementary material for: Modifications to the Aesop's Fable Paradigm Change New Caledonian Crow Performances
Source: PLoS One. 2014 Jul 23;9(7):e103049. doi: 10.1371/journal.pone.0103049 (PMC4108369; doi:10.1371/journal.pone.0103049)
Supplement: File S1 — The percentage of correct choices per bird per trial per experiment. (PDF) [file pone.0103049.s001.pdf]

Bird	Experiment	PercentCorrectChoices	Trial	TrialBin
YG	SandWater	100	1	15
YG	SandWater	75	2	15
YG	SandWater	75	3	15
YG	SandWater	100	4	15
YG	SandWater	100	5	15
YG	SandWater	75	6	610
YG	SandWater	100	7	610
YG	SandWater	100	8	610
YG	SandWater	100	9	610
YG	SandWater	100	10	610
YG	SandWater	100	11	1115
YG	SandWater	100	12	1115
YG	SandWater	100	13	1115
YG	SandWater	100	14	1115
YG	SandWater	100	15	1115
YG	SandWater	100	16	1620
YG	SandWater	100	17	1620
YG	SandWater	100	18	1620
YG	SandWater	100	19	1620
YG	SandWater	100	20	1620
Y	SandWater	25	1	15
Y	SandWater	100	2	15
Y	SandWater	100	3	15
Y	SandWater	0	4	15
Y	SandWater	67	5	15
Y	SandWater	67	6	610
Y	SandWater	100	7	610
Y	SandWater	100	8	610
Y	SandWater	25	9	610
Y	SandWater	100	10	610
Y	SandWater	100	11	1115
Y	SandWater	25	12	1115

Bird	Experiment	PercentCorrectChoices	Trial	TrialBin
Y	SandWater	100	13	1115
Y	SandWater	100	14	1115
Y	SandWater	75	15	1115
Y	SandWater	100	16	1620
Y	SandWater	67	17	1620
Y	SandWater	100	18	1620
Y	SandWater	100	19	1620
Y	SandWater	100	20	1620
WR	SandWater	100	1	15
WR	SandWater	100	2	15
WR	SandWater	100	3	15
WR	SandWater	100	4	15
WR	SandWater	100	5	15
WR	SandWater	50	6	610
WR	SandWater	75	7	610
WR	SandWater	100	8	610
WR	SandWater	50	9	610
WR	SandWater	50	10	610
WR	SandWater	100	11	1115
WR	SandWater	100	12	1115
WR	SandWater	50	13	1115
WR	SandWater	100	14	1115
WR	SandWater	100	15	1115
WR	SandWater	33	16	1620
WR	SandWater	50	17	1620
WR	SandWater	100	18	1620
WR	SandWater	100	19	1620
WR	SandWater	100	20	1620
YR	SandWater	100	1	15
YR	SandWater	0	2	15
YR	SandWater	0	3	15
YR	SandWater	33	4	15

Bird	Experiment	PercentCorrectChoices	Trial	TrialBin
YR	SandWater	100	5	15
YR	SandWater	100	6	610
YR	SandWater	50	7	610
YR	SandWater	100	8	610
YR	SandWater	100	9	610
YR	SandWater	75	10	610
YR	SandWater	100	11	1115
YR	SandWater	67	12	1115
YR	SandWater	67	13	1115
YR	SandWater	100	14	1115
YR	SandWater	25	15	1115
YR	SandWater	100	16	1620
YR	SandWater	100	17	1620
YR	SandWater	100	18	1620
YR	SandWater	100	19	1620
YR	SandWater	100	20	1620
WLB	SandWater	100	1	15
WLB	SandWater	75	2	15
WLB	SandWater	100	3	15
WLB	SandWater	50	4	15
WLB	SandWater	100	5	15
WLB	SandWater	50	6	610
WLB	SandWater	67	7	610
WLB	SandWater	33	8	610
WLB	SandWater	100	9	610
WLB	SandWater	40	10	610
WLB	SandWater	50	11	1115
WLB	SandWater	0	12	1115
WLB	SandWater	0	13	1115
WLB	SandWater	100	14	1115
WLB	SandWater	0	15	1115
WLB	SandWater	100	16	1620

Bird	Experiment	PercentCorrectChoices	Trial	TrialBin
WLB	SandWater	33	17	1620
WLB	SandWater	40	18	1620
WLB	SandWater	50	19	1620
WLB	SandWater	50	20	1620
YG	SinkFloat	50	1	15
YG	SinkFloat	67	2	15
YG	SinkFloat	100	3	15
YG	SinkFloat	67	4	15
YG	SinkFloat	100	5	15
YG	SinkFloat	75	6	610
YG	SinkFloat	50	7	610
YG	SinkFloat	60	8	610
YG	SinkFloat	67	9	610
YG	SinkFloat	60	10	610
YG	SinkFloat	50	11	1115
YG	SinkFloat	100	12	1115
YG	SinkFloat	75	13	1115
YG	SinkFloat	100	14	1115
YG	SinkFloat	100	15	1115
YG	SinkFloat	100	16	1620
YG	SinkFloat	67	17	1620
YG	SinkFloat	100	18	1620
YG	SinkFloat	67	19	1620
YG	SinkFloat	100	20	1620
Y	SinkFloat	67	1	15
Y	SinkFloat	50	2	15
Y	SinkFloat	60	3	15
Y	SinkFloat	100	4	15
Y	SinkFloat	33	5	15
Y	SinkFloat	33	6	610
Y	SinkFloat	33	7	610
Y	SinkFloat	63	8	610

Bird	Experiment	PercentCorrectChoices	Trial	TrialBin
Y	SinkFloat	67	9	610
Y	SinkFloat	83	10	610
Y	SinkFloat	66	11	1115
Y	SinkFloat	100	12	1115
Y	SinkFloat	75	13	1115
Y	SinkFloat	75	14	1115
Y	SinkFloat	100	15	1115
Y	SinkFloat	100	16	1620
Y	SinkFloat	100	17	1620
Y	SinkFloat	100	18	1620
Y	SinkFloat	100	19	1620
Y	SinkFloat	75	20	1620
WR	SinkFloat	71	1	15
WR	SinkFloat	100	2	15
WR	SinkFloat	100	3	15
WR	SinkFloat	57	4	15
WR	SinkFloat	67	5	15
WR	SinkFloat	80	6	610
WR	SinkFloat	67	7	610
WR	SinkFloat	100	8	610
WR	SinkFloat	100	9	610
WR	SinkFloat	100	10	610
WR	SinkFloat	100	11	1115
WR	SinkFloat	100	12	1115
WR	SinkFloat	100	13	1115
WR	SinkFloat	100	14	1115
WR	SinkFloat	100	15	1115
WR	SinkFloat	100	16	1620
WR	SinkFloat	100	17	1620
WR	SinkFloat	100	18	1620
WR	SinkFloat	100	19	1620
WR	SinkFloat	100	20	1620

Bird	Experiment	PercentCorrectChoices	Trial	TrialBin
YR	SinkFloat	75	1	15
YR	SinkFloat	71	2	15
YR	SinkFloat	75	3	15
YR	SinkFloat	100	4	15
YR	SinkFloat	100	5	15
YR	SinkFloat	100	6	610
YR	SinkFloat	100	7	610
YR	SinkFloat	100	8	610
YR	SinkFloat	100	9	610
YR	SinkFloat	100	10	610
YR	SinkFloat	100	11	1115
YR	SinkFloat	100	12	1115
YR	SinkFloat	100	13	1115
YR	SinkFloat	100	14	1115
YR	SinkFloat	100	15	1115
YR	SinkFloat	100	16	1620
YR	SinkFloat	100	17	1620
YR	SinkFloat	100	18	1620
YR	SinkFloat	100	19	1620
YR	SinkFloat	100	20	1620
WLB	SinkFloat	100	1	15
WLB	SinkFloat	100	2	15
WLB	SinkFloat	100	3	15
WLB	SinkFloat	100	4	15
WLB	SinkFloat	100	5	15
WLB	SinkFloat	100	6	610
WLB	SinkFloat	100	7	610
WLB	SinkFloat	100	8	610
WLB	SinkFloat	100	9	610
WLB	SinkFloat	100	10	610
WLB	SinkFloat	100	11	1115
WLB	SinkFloat	100	12	1115

Bird	Experiment	PercentCorrectChoices	Trial	TrialBin
WLB	SinkFloat	100	13	1115
WLB	SinkFloat	100	14	1115
WLB	SinkFloat	100	15	1115
WLB	SinkFloat	100	16	1620
WLB	SinkFloat	100	17	1620
WLB	SinkFloat	100	18	1620
WLB	SinkFloat	100	19	1620
WLB	SinkFloat	100	20	1620
OO	SinkFloat	100	1	15
OO	SinkFloat	100	2	15
OO	SinkFloat	100	3	15
OO	SinkFloat	100	4	15
OO	SinkFloat	100	5	15
OO	SinkFloat	100	6	610
OO	SinkFloat	100	7	610
OO	SinkFloat	100	8	610
OO	SinkFloat	100	9	610
OO	SinkFloat	100	10	610
OO	SinkFloat	100	11	1115
OO	SinkFloat	100	12	1115
OO	SinkFloat	100	13	1115
OO	SinkFloat	100	14	1115
OO	SinkFloat	100	15	1115
OO	SinkFloat	100	16	1620
OO	SinkFloat	100	17	1620
OO	SinkFloat	100	18	1620
OO	SinkFloat	100	19	1620
OO	SinkFloat	100	20	1620
YG	SolidHollow	100	1	15
YG	SolidHollow	100	2	15
YG	SolidHollow	100	3	15
YG	SolidHollow	100	4	15

Bird	Experiment	PercentCorrectChoices	Trial	TrialBin
YG	SolidHollow	100	5	15
YG	SolidHollow	100	6	610
YG	SolidHollow	100	7	610
YG	SolidHollow	67	8	610
YG	SolidHollow	100	9	610
YG	SolidHollow	40	10	610
YG	SolidHollow	50	11	1115
YG	SolidHollow	100	12	1115
YG	SolidHollow	100	13	1115
YG	SolidHollow	100	14	1115
YG	SolidHollow	67	15	1115
YG	SolidHollow	50	16	1620
YG	SolidHollow	100	17	1620
YG	SolidHollow	50	18	1620
YG	SolidHollow	100	19	1620
YG	SolidHollow	100	20	1620
Y	SolidHollow	100	1	15
Y	SolidHollow	100	2	15
Y	SolidHollow	100	3	15
Y	SolidHollow	100	4	15
Y	SolidHollow	100	5	15
Y	SolidHollow	100	6	610
Y	SolidHollow	100	7	610
Y	SolidHollow	100	8	610
Y	SolidHollow	100	9	610
Y	SolidHollow	100	10	610
Y	SolidHollow	100	11	1115
Y	SolidHollow	100	12	1115
Y	SolidHollow	100	13	1115
Y	SolidHollow	100	14	1115
Y	SolidHollow	100	15	1115
Y	SolidHollow	100	16	1620

Bird	Experiment	PercentCorrectChoices	Trial	TrialBin
Y	SolidHollow	100	17	1620
Y	SolidHollow	100	18	1620
Y	SolidHollow	100	19	1620
Y	SolidHollow	100	20	1620
WR	SolidHollow	100	1	15
WR	SolidHollow	100	2	15
WR	SolidHollow	100	3	15
WR	SolidHollow	100	4	15
WR	SolidHollow	100	5	15
WR	SolidHollow	100	6	610
WR	SolidHollow	100	7	610
WR	SolidHollow	100	8	610
WR	SolidHollow	100	9	610
WR	SolidHollow	100	10	610
WR	SolidHollow	100	11	1115
WR	SolidHollow	100	12	1115
WR	SolidHollow	100	13	1115
WR	SolidHollow	100	14	1115
WR	SolidHollow	100	15	1115
WR	SolidHollow	100	16	1620
WR	SolidHollow	100	17	1620
WR	SolidHollow	100	18	1620
WR	SolidHollow	100	19	1620
WR	SolidHollow	100	20	1620
YR	SolidHollow	100	1	15
YR	SolidHollow	100	2	15
YR	SolidHollow	100	3	15
YR	SolidHollow	100	4	15
YR	SolidHollow	100	5	15
YR	SolidHollow	100	6	610
YR	SolidHollow	100	7	610
YR	SolidHollow	100	8	610

Bird	Experiment	PercentCorrectChoices	Trial	TrialBin
YR	SolidHollow	100	9	610
YR	SolidHollow	100	10	610
YR	SolidHollow	100	11	1115
YR	SolidHollow	100	12	1115
YR	SolidHollow	100	13	1115
YR	SolidHollow	100	14	1115
YR	SolidHollow	100	15	1115
YR	SolidHollow	100	16	1620
YR	SolidHollow	100	17	1620
YR	SolidHollow	100	18	1620
YR	SolidHollow	100	19	1620
YR	SolidHollow	100	20	1620
WLB	SolidHollow	100	1	15
WLB	SolidHollow	100	2	15
WLB	SolidHollow	100	3	15
WLB	SolidHollow	100	4	15
WLB	SolidHollow	100	5	15
WLB	SolidHollow	100	6	610
WLB	SolidHollow	100	7	610
WLB	SolidHollow	100	8	610
WLB	SolidHollow	100	9	610
WLB	SolidHollow	100	10	610
WLB	SolidHollow	67	11	1115
WLB	SolidHollow	100	12	1115
WLB	SolidHollow	100	13	1115
WLB	SolidHollow	100	14	1115
WLB	SolidHollow	67	15	1115
WLB	SolidHollow	100	16	1620
WLB	SolidHollow	100	17	1620
WLB	SolidHollow	100	18	1620
WLB	SolidHollow	100	19	1620
WLB	SolidHollow	100	20	1620

Bird	Experiment	PercentCorrectChoices	Trial	TrialBin
OO	SolidHollow	100	1	15
OO	SolidHollow	100	2	15
OO	SolidHollow	100	3	15
OO	SolidHollow	100	4	15
OO	SolidHollow	100	5	15
OO	SolidHollow	100	6	610
OO	SolidHollow	100	7	610
OO	SolidHollow	100	8	610
OO	SolidHollow	100	9	610
OO	SolidHollow	100	10	610
OO	SolidHollow	100	11	1115
OO	SolidHollow	100	12	1115
OO	SolidHollow	100	13	1115
OO	SolidHollow	100	14	1115
OO	SolidHollow	100	15	1115
OO	SolidHollow	100	16	1620
OO	SolidHollow	100	17	1620
OO	SolidHollow	100	18	1620
OO	SolidHollow	100	19	1620
OO	SolidHollow	100	20	1620
YG	WideNarrowEqual	100	1	15
YG	WideNarrowEqual	100	2	15
YG	WideNarrowEqual	50	3	15
YG	WideNarrowEqual	0	4	15
YG	WideNarrowEqual	50	5	15
YG	WideNarrowEqual	25	6	610
YG	WideNarrowEqual	100	7	610
YG	WideNarrowEqual	75	8	610
YG	WideNarrowEqual	100	9	610
YG	WideNarrowEqual	100	10	610
YG	WideNarrowEqual	100	11	1115
YG	WideNarrowEqual	100	12	1115

Bird	Experiment	PercentCorrectChoices	Trial	TrialBin
YG	WideNarrowEqual	67	13	1115
YG	WideNarrowEqual	100	14	1115
YG	WideNarrowEqual	50	15	1115
YG	WideNarrowEqual	100	16	1620
YG	WideNarrowEqual	100	17	1620
YG	WideNarrowEqual	100	18	1620
YG	WideNarrowEqual	100	19	1620
YG	WideNarrowEqual	67	20	1620
Y	WideNarrowEqual	0	1	15
Y	WideNarrowEqual	100	2	15
Y	WideNarrowEqual	67	3	15
Y	WideNarrowEqual	67	4	15
Y	WideNarrowEqual	50	5	15
Y	WideNarrowEqual	100	6	610
Y	WideNarrowEqual	0	7	610
Y	WideNarrowEqual	25	8	610
Y	WideNarrowEqual	100	9	610
Y	WideNarrowEqual	67	10	610
Y	WideNarrowEqual	100	11	1115
Y	WideNarrowEqual	100	12	1115
Y	WideNarrowEqual	50	13	1115
Y	WideNarrowEqual	50	14	1115
Y	WideNarrowEqual	100	15	1115
Y	WideNarrowEqual	0	16	1620
Y	WideNarrowEqual	100	17	1620
Y	WideNarrowEqual	25	18	1620
Y	WideNarrowEqual	100	19	1620
Y	WideNarrowEqual	0	20	1620
WR	WideNarrowEqual	67	1	15
WR	WideNarrowEqual	100	2	15
WR	WideNarrowEqual	33	3	15
WR	WideNarrowEqual	67	4	15

Bird	Experiment	PercentCorrectChoices	Trial	TrialBin
WR	WideNarrowEqual	100	5	15
WR	WideNarrowEqual	50	6	610
WR	WideNarrowEqual	50	7	610
WR	WideNarrowEqual	0	8	610
WR	WideNarrowEqual	100	9	610
WR	WideNarrowEqual	100	10	610
WR	WideNarrowEqual	100	11	1115
WR	WideNarrowEqual	67	12	1115
WR	WideNarrowEqual	100	13	1115
WR	WideNarrowEqual	100	14	1115
WR	WideNarrowEqual	100	15	1115
WR	WideNarrowEqual	50	16	1620
WR	WideNarrowEqual	100	17	1620
WR	WideNarrowEqual	100	18	1620
WR	WideNarrowEqual	67	19	1620
WR	WideNarrowEqual	0	20	1620
YR	WideNarrowEqual	50	1	15
YR	WideNarrowEqual	50	2	15
YR	WideNarrowEqual	50	3	15
YR	WideNarrowEqual	67	4	15
YR	WideNarrowEqual	67	5	15
YR	WideNarrowEqual	67	6	610
YR	WideNarrowEqual	75	7	610
YR	WideNarrowEqual	25	8	610
YR	WideNarrowEqual	25	9	610
YR	WideNarrowEqual	50	10	610
YR	WideNarrowEqual	67	11	1115
YR	WideNarrowEqual	100	12	1115
YR	WideNarrowEqual	100	13	1115
YR	WideNarrowEqual	50	14	1115
YR	WideNarrowEqual	100	15	1115
YR	WideNarrowEqual	75	16	1620

Bird	Experiment	PercentCorrectChoices	Trial	TrialBin
YR	WideNarrowEqual	100	17	1620
YR	WideNarrowEqual	100	18	1620
YR	WideNarrowEqual	67	19	1620
YR	WideNarrowEqual	100	20	1620
WLB	WideNarrowEqual	25	1	15
WLB	WideNarrowEqual	50	2	15
WLB	WideNarrowEqual	100	3	15
WLB	WideNarrowEqual	0	4	15
WLB	WideNarrowEqual	100	5	15
WLB	WideNarrowEqual	100	6	610
WLB	WideNarrowEqual	0	7	610
WLB	WideNarrowEqual	100	8	610
WLB	WideNarrowEqual	25	9	610
WLB	WideNarrowEqual	67	10	610
WLB	WideNarrowEqual	100	11	1115
WLB	WideNarrowEqual	25	12	1115
WLB	WideNarrowEqual	25	13	1115
WLB	WideNarrowEqual	100	14	1115
WLB	WideNarrowEqual	100	15	1115
WLB	WideNarrowEqual	67	16	1620
WLB	WideNarrowEqual	100	17	1620
WLB	WideNarrowEqual	25	18	1620
WLB	WideNarrowEqual	100	19	1620
WLB	WideNarrowEqual	75	20	1620
OO	WideNarrowEqual	50	1	15
OO	WideNarrowEqual	67	2	15
OO	WideNarrowEqual	100	3	15
OO	WideNarrowEqual	100	4	15
OO	WideNarrowEqual	67	5	15
OO	WideNarrowEqual	0	6	610
OO	WideNarrowEqual	100	7	610
OO	WideNarrowEqual	67	8	610

Bird	Experiment	PercentCorrectChoices	Trial	TrialBin
OO	WideNarrowEqual	100	9	610
OO	WideNarrowEqual	100	10	610
OO	WideNarrowEqual	100	11	1115
OO	WideNarrowEqual	67	12	1115
OO	WideNarrowEqual	50	13	1115
OO	WideNarrowEqual	100	14	1115
OO	WideNarrowEqual	75	15	1115
OO	WideNarrowEqual	100	16	1620
OO	WideNarrowEqual	100	17	1620
OO	WideNarrowEqual	100	18	1620
OO	WideNarrowEqual	100	19	1620
OO	WideNarrowEqual	100	20	1620
YG	WideNarrowUnequal	0	1	15
YG	WideNarrowUnequal	75	2	15
YG	WideNarrowUnequal	75	3	15
YG	WideNarrowUnequal	100	4	15
YG	WideNarrowUnequal	100	5	15
YG	WideNarrowUnequal	0	6	610
YG	WideNarrowUnequal	25	7	610
YG	WideNarrowUnequal	75	8	610
YG	WideNarrowUnequal	100	9	610
YG	WideNarrowUnequal	0	10	610
YG	WideNarrowUnequal	100	11	1115
YG	WideNarrowUnequal	75	12	1115
YG	WideNarrowUnequal	100	13	1115
YG	WideNarrowUnequal	100	14	1115
YG	WideNarrowUnequal	100	15	1115
YG	WideNarrowUnequal	25	16	1620
YG	WideNarrowUnequal	75	17	1620
YG	WideNarrowUnequal	100	18	1620
YG	WideNarrowUnequal	100	19	1620
YG	WideNarrowUnequal	100	20	1620

Bird	Experiment	PercentCorrectChoices	Trial	TrialBin
WR	WideNarrowUnequal	25	1	15
WR	WideNarrowUnequal	0	2	15
WR	WideNarrowUnequal	100	3	15
WR	WideNarrowUnequal	0	4	15
WR	WideNarrowUnequal	33	5	15
WR	WideNarrowUnequal	50	6	610
WR	WideNarrowUnequal	100	7	610
WR	WideNarrowUnequal	33	8	610
WR	WideNarrowUnequal	50	9	610
WR	WideNarrowUnequal	33	10	610
WR	WideNarrowUnequal	75	11	1115
WR	WideNarrowUnequal	100	12	1115
WR	WideNarrowUnequal	50	13	1115
WR	WideNarrowUnequal	50	14	1115
WR	WideNarrowUnequal	25	15	1115
WR	WideNarrowUnequal	100	16	1620
WR	WideNarrowUnequal	100	17	1620
WR	WideNarrowUnequal	50	18	1620
WR	WideNarrowUnequal	67	19	1620
WR	WideNarrowUnequal	100	20	1620
YR	WideNarrowUnequal	100	1	15
YR	WideNarrowUnequal	75	2	15
YR	WideNarrowUnequal	75	3	15
YR	WideNarrowUnequal	100	4	15
YR	WideNarrowUnequal	100	5	15
YR	WideNarrowUnequal	100	6	610
YR	WideNarrowUnequal	100	7	610
YR	WideNarrowUnequal	100	8	610
YR	WideNarrowUnequal	100	9	610
YR	WideNarrowUnequal	100	10	610
YR	WideNarrowUnequal	100	11	1115
YR	WideNarrowUnequal	100	12	1115

Bird	Experiment	PercentCorrectChoices	Trial	TrialBin
YR	WideNarrowUnequal	100	13	1115
YR	WideNarrowUnequal	100	14	1115
YR	WideNarrowUnequal	100	15	1115
YR	WideNarrowUnequal	100	16	1620
YR	WideNarrowUnequal	100	17	1620
YR	WideNarrowUnequal	50	18	1620
YR	WideNarrowUnequal	100	19	1620
YR	WideNarrowUnequal	100	20	1620
OO	WideNarrowUnequal	100	1	15
OO	WideNarrowUnequal	100	2	15
OO	WideNarrowUnequal	100	3	15
OO	WideNarrowUnequal	100	4	15
OO	WideNarrowUnequal	100	5	15
OO	WideNarrowUnequal	100	6	610
OO	WideNarrowUnequal	100	7	610
OO	WideNarrowUnequal	100	8	610
OO	WideNarrowUnequal	100	9	610
OO	WideNarrowUnequal	100	10	610
OO	WideNarrowUnequal	100	11	1115
OO	WideNarrowUnequal	100	12	1115
OO	WideNarrowUnequal	100	13	1115
OO	WideNarrowUnequal	100	14	1115
OO	WideNarrowUnequal	100	15	1115
OO	WideNarrowUnequal	100	16	1620
OO	WideNarrowUnequal	100	17	1620
OO	WideNarrowUnequal	100	18	1620
OO	WideNarrowUnequal	100	19	1620
OO	WideNarrowUnequal	100	20	1620
YG	ColoredU-tube	75	1	15
YG	ColoredU-tube	100	2	15
YG	ColoredU-tube	50	3	15
YG	ColoredU-tube	0	4	15

Bird	Experiment	PercentCorrectChoices	Trial	TrialBin
YG	ColoredU-tube	0	5	15
YG	ColoredU-tube	100	6	610
YG	ColoredU-tube	0	7	610
YG	ColoredU-tube	50	8	610
YG	ColoredU-tube	100	9	610
YG	ColoredU-tube	100	10	610
YG	ColoredU-tube	0	11	1115
YG	ColoredU-tube	100	12	1115
YG	ColoredU-tube	75	13	1115
YG	ColoredU-tube	100	14	1115
YG	ColoredU-tube	0	15	1115
YG	ColoredU-tube	100	16	1620
YG	ColoredU-tube	100	17	1620
YG	ColoredU-tube	0	18	1620
YG	ColoredU-tube	0	19	1620
YG	ColoredU-tube	50	20	1620
Y	ColoredU-tube	25	1	15
Y	ColoredU-tube	100	2	15
Y	ColoredU-tube	0	3	15
Y	ColoredU-tube	100	4	15
Y	ColoredU-tube	50	5	15
Y	ColoredU-tube	100	6	610
Y	ColoredU-tube	0	7	610
Y	ColoredU-tube	50	8	610
Y	ColoredU-tube	67	9	610
Y	ColoredU-tube	0	10	610
Y	ColoredU-tube	0	11	1115
Y	ColoredU-tube	75	12	1115
Y	ColoredU-tube	25	13	1115
Y	ColoredU-tube	25	14	1115
Y	ColoredU-tube	100	15	1115
Y	ColoredU-tube	0	16	1620

Bird	Experiment	PercentCorrectChoices	Trial	TrialBin
Y	ColoredU-tube	100	17	1620
Y	ColoredU-tube	0	18	1620
Y	ColoredU-tube	100	19	1620
Y	ColoredU-tube	0	20	1620
WR	ColoredU-tube	0	1	15
WR	ColoredU-tube	100	2	15
WR	ColoredU-tube	67	3	15
WR	ColoredU-tube	100	4	15
WR	ColoredU-tube	50	5	15
WR	ColoredU-tube	100	6	610
WR	ColoredU-tube	100	7	610
WR	ColoredU-tube	75	8	610
WR	ColoredU-tube	100	9	610
WR	ColoredU-tube	100	10	610
WR	ColoredU-tube	0	11	1115
WR	ColoredU-tube	100	12	1115
WR	ColoredU-tube	0	13	1115
WR	ColoredU-tube	0	14	1115
WR	ColoredU-tube	100	15	1115
WR	ColoredU-tube	100	16	1620
WR	ColoredU-tube	100	17	1620
WR	ColoredU-tube	100	18	1620
WR	ColoredU-tube	75	19	1620
WR	ColoredU-tube	100	20	1620
YR	ColoredU-tube	100	1	15
YR	ColoredU-tube	100	2	15
YR	ColoredU-tube	0	3	15
YR	ColoredU-tube	0	4	15
YR	ColoredU-tube	33	5	15
YR	ColoredU-tube	25	6	610
YR	ColoredU-tube	100	7	610
YR	ColoredU-tube	25	8	610

Bird	Experiment	PercentCorrectChoices	Trial	TrialBin
YR	ColoredU-tube	25	9	610
YR	ColoredU-tube	100	10	610
YR	ColoredU-tube	100	11	1115
YR	ColoredU-tube	25	12	1115
YR	ColoredU-tube	50	13	1115
YR	ColoredU-tube	100	14	1115
YR	ColoredU-tube	0	15	1115
YR	ColoredU-tube	75	16	1620
YR	ColoredU-tube	100	17	1620
YR	ColoredU-tube	25	18	1620
YR	ColoredU-tube	100	19	1620
YR	ColoredU-tube	0	20	1620
WLB	ColoredU-tube	100	1	15
WLB	ColoredU-tube	0	2	15
WLB	ColoredU-tube	0	3	15
WLB	ColoredU-tube	0	4	15
WLB	ColoredU-tube	50	5	15
WLB	ColoredU-tube	100	6	610
WLB	ColoredU-tube	0	7	610
WLB	ColoredU-tube	100	8	610
WLB	ColoredU-tube	50	9	610
WLB	ColoredU-tube	0	10	610
WLB	ColoredU-tube	100	11	1115
WLB	ColoredU-tube	0	12	1115
WLB	ColoredU-tube	100	13	1115
WLB	ColoredU-tube	100	14	1115
WLB	ColoredU-tube	0	15	1115
WLB	ColoredU-tube	100	16	1620
WLB	ColoredU-tube	0	17	1620
WLB	ColoredU-tube	0	18	1620
WLB	ColoredU-tube	50	19	1620
WLB	ColoredU-tube	67	20	1620

Bird	Experiment	PercentCorrectChoices	Trial	TrialBin
YG	UncoveredU-tube	0	1	15
YG	UncoveredU-tube	0	2	15
YG	UncoveredU-tube	0	3	15
YG	UncoveredU-tube	100	4	15
YG	UncoveredU-tube	67	5	15
YG	UncoveredU-tube	25	6	610
YG	UncoveredU-tube	50	7	610
YG	UncoveredU-tube	25	8	610
YG	UncoveredU-tube	50	9	610
YG	UncoveredU-tube	25	10	610
YG	UncoveredU-tube	0	11	1115
YG	UncoveredU-tube	100	12	1115
YG	UncoveredU-tube	100	13	1115
YG	UncoveredU-tube	0	14	1115
YG	UncoveredU-tube	67	15	1115
YG	UncoveredU-tube	0	16	1620
YG	UncoveredU-tube	33	17	1620
YG	UncoveredU-tube	100	18	1620
YG	UncoveredU-tube	0	19	1620
YG	UncoveredU-tube	0	20	1620
Y	UncoveredU-tube	0	1	15
Y	UncoveredU-tube	100	2	15
Y	UncoveredU-tube	25	3	15
Y	UncoveredU-tube	67	4	15
Y	UncoveredU-tube	100	5	15
Y	UncoveredU-tube	50	6	610
Y	UncoveredU-tube	100	7	610
Y	UncoveredU-tube	0	8	610
Y	UncoveredU-tube	100	9	610
Y	UncoveredU-tube	25	10	610
Y	UncoveredU-tube	67	11	1115
Y	UncoveredU-tube	25	12	1115

Bird	Experiment	PercentCorrectChoices	Trial	TrialBin
Y	UncoveredU-tube	100	13	1115
Y	UncoveredU-tube	0	14	1115
Y	UncoveredU-tube	25	15	1115
Y	UncoveredU-tube	100	16	1620
Y	UncoveredU-tube	0	17	1620
Y	UncoveredU-tube	0	18	1620
Y	UncoveredU-tube	100	19	1620
Y	UncoveredU-tube	0	20	1620
WR	UncoveredU-tube	100	1	15
WR	UncoveredU-tube	25	2	15
WR	UncoveredU-tube	50	3	15
WR	UncoveredU-tube	100	4	15
WR	UncoveredU-tube	100	5	15
WR	UncoveredU-tube	0	6	610
WR	UncoveredU-tube	100	7	610
WR	UncoveredU-tube	0	8	610
WR	UncoveredU-tube	50	9	610
WR	UncoveredU-tube	100	10	610
WR	UncoveredU-tube	0	11	1115
WR	UncoveredU-tube	0	12	1115
WR	UncoveredU-tube	100	13	1115
WR	UncoveredU-tube	0	14	1115
WR	UncoveredU-tube	100	15	1115
WR	UncoveredU-tube	100	16	1620
WR	UncoveredU-tube	0	17	1620
WR	UncoveredU-tube	100	18	1620
WR	UncoveredU-tube	0	19	1620
WR	UncoveredU-tube	0	20	1620
YR	UncoveredU-tube	100	1	15
YR	UncoveredU-tube	100	2	15
YR	UncoveredU-tube	50	3	15
YR	UncoveredU-tube	100	4	15

Bird	Experiment	PercentCorrectChoices	Trial	TrialBin
YR	UncoveredU-tube	0	5	15
YR	UncoveredU-tube	100	6	610
YR	UncoveredU-tube	0	7	610
YR	UncoveredU-tube	100	8	610
YR	UncoveredU-tube	100	9	610
YR	UncoveredU-tube	100	10	610
YR	UncoveredU-tube	100	11	1115
YR	UncoveredU-tube	100	12	1115
YR	UncoveredU-tube	0	13	1115
YR	UncoveredU-tube	100	14	1115
YR	UncoveredU-tube	0	15	1115
YR	UncoveredU-tube	0	16	1620
YR	UncoveredU-tube	33	17	1620
YR	UncoveredU-tube	33	18	1620
YR	UncoveredU-tube	0	19	1620
YR	UncoveredU-tube	33	20	1620
WLB	UncoveredU-tube	0	1	15
WLB	UncoveredU-tube	100	2	15
WLB	UncoveredU-tube	25	3	15
WLB	UncoveredU-tube	50	4	15
WLB	UncoveredU-tube	100	5	15
WLB	UncoveredU-tube	75	6	610
WLB	UncoveredU-tube	100	7	610
WLB	UncoveredU-tube	75	8	610
WLB	UncoveredU-tube	100	9	610
WLB	UncoveredU-tube	0	10	610
WLB	UncoveredU-tube	100	11	1115
WLB	UncoveredU-tube	50	12	1115
WLB	UncoveredU-tube	100	13	1115
WLB	UncoveredU-tube	0	14	1115
WLB	UncoveredU-tube	0	15	1115
WLB	UncoveredU-tube	25	16	1620

Bird	Experiment	PercentCorrectChoices	Trial	TrialBin
WLB	UncoveredU-tube	100	17	1620
WLB	UncoveredU-tube	100	18	1620
WLB	UncoveredU-tube	100	19	1620
WLB	UncoveredU-tube	25	20	1620
