# Supplementary material for: Modifications to the Aesop's Fable Paradigm Change New Caledonian Crow Performances
Source: PLoS One. 2014 Jul 23;9(7):e103049. doi: 10.1371/journal.pone.0103049 (PMC4108369; doi:10.1371/journal.pone.0103049)
Supplement: File S2 — The order in which each choice was made for all birds in all trials in all experiments. (PDF) [file pone.0103049.s002.pdf]

Supporting Information 2: Object insertion order across trials per individual

(Logan, Jelbert, Breen, Gray, Taylor. 2014. Modifications to the Aesop's Fable paradigm change performances in New Caledonian crows. PLOS ONE)

Q						
	Insertion Order					
Trial	1	2	3	4	5	6
1	X					
2						
3				X		
4			X			
5	X					
6				X		
7		X				
8	X					
9	X					
10						
11				X		
12				X		
13			X			
14			X			
15			X			
16			X			
17		X				
18		X				
19						
20				X		

007						
	Insertion Order					
Trial	1	2	3	4	5	6
1						
2						
3						
4						
5			X			
6			X			
7	X					
8				X		
9						
10				X		
11						
12						
13			X			
14			X			
15						
16	X					
17			X			
18			X			
19		X				
20			X			

Kitty						
	Insertion Order					
Trial	1	2	3	4	5	6
1			X			
2		X				
3	X					
4	X					
5	X					
6						
7						
8						
9						
10						
11	X					
12	X					
13		X				
14		X				
15						
16			X			
17				X		
18	X					
19	X					
20		X				

Lady						
	Insertion Order					
Trial	1	2	3	4	5	6
1			X			
2						
3						
4						
5			X			
6		X				
7		X				
8			X			
9				X		
10				X		
11			X			
12			X			
13			X			
14			X			
15				X		
16			X			
17				X		
18			X			
19			X			
20		X				

Damien						
	Insertion Order					
Trial	1	2	3	4	5	6
1		X				
2						
3		X				
4			X			
5	X					
6				X		
7			X			
8						
9		X				
10					X	
11						
12						
13						
14	X					
15			X			
16	X					
17	X					
18						
19		X				
20		X				

Table S2.1. Water vs. Sand: order in which stones were inserted (columns) into water (dark gray) and sand (light gray) tubes and whether the bird successfully obtained the food (marked with an X) for trials 1-20 (rows). Note that occasionally food was obtained from the sand tube because a bird’s motivation could change within a trial, therefore changing their reachable distance.

Q								
	Insertion Order							
Trial	1	2	3	4	5	6	7	8
1								
2								
3								
4			X					
5		X						
6								
7	X							
8								
9			X					
10								
11								
12			X					
13								
14	X							
15		X						
16	X							
17			X					
18								
19					X			
20	X							

007								
	Insertion Order							
Trial	1	2	3	4	5	6	7	8
1			X					
2						X		
3				X				
4	X							
5			X					
6	X							
7	X							
8								X
9		X						
10						X		
11			X					
12		X						
13				X				
14				X				
15			X					
16		X						
17	X							
18			X					
19	X							
20			X					

Kitty							
	Insertion Order						
Trial	1	2	3	4	5	6	7
1							X
2		X					
3		X					
4							X
5						X	
6					X		
7			X				
8		X					
9			X				
10	X						
11	X						
12			X				
13			X				
14				X			
15	X						
16		X					
17		X					
18			X				
19			X				
20							

Lady							
	Insertion Order						
Trial	1	2	3	4	5	6	7
1				X			
2						X	
3				X			
4				X			
5				X			
6				X			
7	X						
8			X				
9			X				
10	X						
11			X				
12			X				
13		X					
14				X			
15				X			
16			X				
17			X				
18		X					
19		X					
20		X					

Damien				
	Insertion Order			
Trial	1	2	3	4
1	X			
2		X		
3		X		
4			X	
5			X	
6			X	
7			X	
8		X		
9		X		
10		X		
11			X	
12				X
13			X	
14	X			
15	X			
16		X		
17			X	
18				X
19				X
20			X	

Buster				
	Insertion Order			
Trial	1	2	3	4
1			X	
2				
3			X	
4		X		
5			X	
6			X	
7		X		
8		X		
9			X	
10		X		
11		X		
12			X	
13			X	
14			X	
15			X	
16			X	
17			X	
18				X
19			X	
20		X		

Table S2.2. Sinking vs. Floating: order in which sinking (dark gray) or floating (light gray) objects were inserted (columns) into a water tube and whether the bird successfully obtained the food (marked with an X) for trials 1-20 (rows).

Q					
	Insertion Order				
Trial	1	2	3	4	5
1		X			
2		X			
3		X			
4			X		
5			X		
6			X		
7			X		
8					
9		X			
10					
11					
12	X				
13					
14	X				
15			X		
16		X			
17		X			
18					
19					
20					

007					
	Insertion Order				
Trial	1	2	3	4	5
1		X			
2		X			
3			X		
4		X			
5			X		
6		X			
7		X			
8		X			
9		X			
10		X			
11	X				
12			X		
13		X			
14		X			
15		X			
16			X		
17		X			
18		X			
19	X				
20		X			

Kitty					
	Insertion Order				
Trial	1	2	3	4	5
1		X			
2			X		
3		X			
4	X				
5	X				
6		X			
7			X		
8		X			
9	X				
10		X			
11		X			
12			X		
13	X				
14				X	
15		X			
16			X		
17		X			
18		X			
19		X			
20			X		

Lady					
	Insertion Order				
Trial	1	2	3	4	5
1			X		
2			X		
3		X			
4		X			
5		X			
6		X			
7		X			
8		X			
9		X			
10		X			
11	X				
12	X				
13		X			
14	X				
15	X				
16		X			
17		X			
18			X		
19	X				
20		X			

Damien					
	Insertion Order				
Trial	1	2	3	4	5
1		X			
2		X			
3		X			
4		X			
5		X			
6			X		
7		X			
8	X				
9		X			
10			X		
11			X		
12			X		
13	X				
14		X			
15			X		
16		X			
17	X				
18		X			
19		X			
20		X			

Buster					
	Insertion Order				
Trial	1	2	3	4	5
1		X			
2			X		
3		X			
4		X			
5	X				
6	X				
7		X			
8		X			
9		X			
10			X		
11		X			
12		X			
13			X		
14		X			
15	X				
16		X			
17	X				
18	X				
19		X			
20		X			

Table S2.3. Solid vs. Hollow: order in which solid (dark gray) or hollow (light gray) objects were inserted (columns) into a water tube and whether the bird successfully obtained the food (marked with an X) for trials 1-20 (rows).

Q					007					Kitty					Lady					Damien					Buster				
	Insertion Order					Insertion Order					Insertion Order					Insertion Order					Insertion Order					Insertion Order			
Trial	1	2	3	4	Trial	1	2	3	4	Trial	1	2	3	4	Trial	1	2	3	4	Trial	1	2	3	4	Trial	1	2	3	4
1					1					1			X		1		X			1					1				X
2		X			2	X				2		X			2		X			2	X				2			X	
3				X	3			X		3			X		3		X			3	X				3		X		
4					4			X		4			X		4			X		4			X		4		X		
5				X	5				X	5		X			5			X		5	X				5			X	
6					6			X		6				X	6			X		6	X				6				
7					7					7				X	7				X	7					7		X		
8				X	8					8					8	X				8	X				8			X	
9		X			9		X			9		X			9					9					9		X		
10			X		10			X		10		X			10				X	10		X			10		X		
11			X		11	X				11		X			11			X		11	X				11		X		
12		X			12	X				12			X		12			X		12					12			X	
13			X		13		X			13		X			13			X		13					13		X		
14		X			14				X	14		X			14				X	14		X			14		X		
15				X	15		X			15		X			15		X			15		X			15				X
16	X				16					16				X	16				X	16			X		16			X	
17			X		17			X		17		X			17		X			17		X			17				X
18		X			18					18		X			18		X			18				X	18			X	
19		X			19			X		19			X		19			X		19		X			19		X		
20			X		20					20					20			X		20				X	20			X	

Table S2.4. Narrow vs. Wide Equal Water Levels: order in which objects were inserted (columns) into narrow (dark gray; functional) or wide (light gray; non-functional) water tubes and whether the bird successfully obtained the food (marked with an X) for trials 1-20 (rows). Note that Damien obtained food from the wide tube once because his motivation changed unexpectedly within that trial, therefore changing his reachable distance.

Q					Kitty					Lady					Buster				
	Insertion Order					Insertion Order					Insertion Order					Insertion Order			
**Trial**	1	2	3	4	**Trial**	1	2	3	4	**Trial**	1	2	3	4	**Trial**	1	2	3	4
1					1					1	X				1		X		
2					2					2				X	2			X	
3				X	3					3					3	X			
4					4					4	X				4		X		
5		X			5					5		X			5			X	
6					6				X	6		X			6				X
7					7			X		7			X		7				X
8					8			X		8	X				8				X
9				X	9	X				9		X			9	X			
10					10					10				X	10		X		
11	X				11				X	11	X				11		X		
12				X	12		X			12				X	12			X	
13				X	13		X			13	X				13			X	
14		X			14					14			X		14				X
15		X			15				X	15		X			15			X	
16					16	X				16	X				16				X
17				X	17	X				17	X				17				X
18				X	18				X	18					18		X		
19			X		19			X		19		X			19		X		
20					20				X	20				X	20		X		

Table S2.5. Narrow vs. Wide Unequal Water Levels: order in which objects were inserted (columns) into wide (dark gray; functional) or narrow (light gray; non-functional) water tubes and whether the bird successfully obtained the food (marked with an X) for trials 1-20 (rows).

007					Q					Kitty					Lady					Damien				
	Insertion Order					Insertion Order					Insertion Order					Insertion Order					Insertion Order			
**Trial**	1	2	3	4	**Trial**	1	2	3	4	**Trial**	1	2	3	4	**Trial**	1	2	3	4	**Trial**	1	2	3	4
1					1				X	1					1		X			1			X	
2		X			2					2			X		2	X				2				
3					3		X			3			X		3					3				
4	X				4		X			4		X			4					4				
5				X	5					5				X	5					5		X		
6	X				6					6	X				6					6		X		
7					7					7	X				7			X		7				
8				X	8					8				X	8					8	X			
9			X		9		X			9			X		9					9				X
10					10		X			10			X		10			X		10				
11					11					11					11		X			11	X			
12				X	12		X			12				X	12				X	12				
13					13				X	13					13				X	13		X		
14					14	X				14					14		X			14	X			
15			X		15					15	X				15					15				
16					16		X			16		X			16					16	X			
17			X		17				X	17		X			17		X			17				
18					18					18		X			18				X	18				
19					19					19				X	19	X				19				X
20					20					20		X			20					20			X	

Table S2.6. Colored U-tube: order in which stones were inserted (columns) into connected (dark gray; functional) or unconnected (light gray; non-functional) wide water tubes and whether the bird successfully obtained the food (marked with an X) for trials 1-20 (rows). Note that Q obtained food from the unconnected tube once because his motivation changed unexpectedly within that trial, therefore changing his reachable distance.

007					Kitty					Q					Lady					Damien				
	Insertion Order					Insertion Order					Insertion Order					Insertion Order					Insertion Order			
**Trial**	1	2	3	4	**Trial**	1	2	3	4	**Trial**	1	2	3	4	**Trial**	1	2	3	4	**Trial**	1	2	3	4
1					1				X	1					1	X				1				
2			X		2				X	2					2			X		2			X	
3					3					3					3					3				X
4			X		4			X		4		X			4				X	4		X		
5			X		5		X			5			X		5					5		X		
6				X	6					6					6	X				6				X
7	X				7	X				7				X	7					7	X			
8	X				8					8					8	X				8				X
9			X		9					9				X	9		X			9		X		
10					10		X			10					10	X				10				
11			X		11					11					11	X				11				
12				X	12					12			X		12	X				12				X
13		X			13				X	13		X			13					13	X			
14					14					14					14		X			14				
15					15		X			15			X		15					15				
16	X				16		X			16					16					16				
17					17					17			X		17	X				17	X			
18					18		X			18	X				18			X		18		X		
19		X			19					19					19					19		X		
20					20	!				20					20			X		20				

Table S2.7. Uncovered U-tube: order in which stones were inserted (columns) into connected (dark gray; functional) or unconnected (light gray; non-functional) wide water tubes and whether the bird successfully obtained the food (marked with an X; marked with an ! when obtained from the unconnected tube due to changing motivation levels and thus reachable distance within a trial) for trials 1-20 (rows). Note that 007 obtained food from the connected tube once without dropping objects into the correct wide tube because his motivation changed unexpectedly within that trial, therefore changing his reachable distance.

007							Kitty					Trooper					Lady					Damien				
	Insertion Order							Insertion Order					Insertion Order					Insertion Order					Insertion Order			
**Trial**	1	2	3	4	5	6	**Trial**	1	2	3	4	**Trial**	1	2	3	4	**Trial**	1	2	3	4	**Trial**	1	2	3	4
1			X				1		X			1	X				1		X			1	X			
2						X	2		X			2		X			2		X			2	X			
3		X					3		X			3			X		3		X			3		X		
4		X					4		X			4		X			4		X			4			X	
5			X				5		X			5	X				5		X			5	X			
6		X					6		X			6	X				6		X			6		X		
7		X					7			X		7		X			7		X			7		X		
8	X						8			X		8		X			8			X		8		X		
9		X					9		X			9		X			9		X			9	X			
10		X					10		X			10		X			10			X		10		X		
11		X					11		X			11	X				11			X		11			X	
12		X					12		X			12		X			12		X			12				X
13		X					13		X			13		X			13			X		13			X	
14		X					14			X		14	X				14		X			14			X	
15		X					15				X	15		X			15		X			15	X			
16		X					16		X			16		X			16			X		16	X			
17		X					17			X		17		X			17		X			17			X	
18			X				18		X			18			X		18		X			18		X		
19		X					19			X		19		X			19		X			19	X			
20		X					20		X			20		X			20		X			20		X		

Table S2.8. Solid vs. Hollow Platform: order in which solid (dark gray) or hollow (light gray) objects were inserted (columns) onto the collapsible platform for trials 1-20 (rows). Note that Damien obtained food by dropping a hollow object into the tube because his motivation changed unexpectedly within that trial, therefore changing his reachable distance.
